# Exploration of the shared gene signatures and molecular mechanisms between cardioembolic stroke and ischemic stroke

**DOI:** 10.3389/fneur.2025.1567902

**Published:** 2025-04-08

**Authors:** Xuan Wang, Xueyuan Liu

**Affiliations:** ^1^Department of Neurology, Shanghai Tenth People's Hospital, Tongji University School of Medicine, Shanghai, China; ^2^School of Medicine, Tongji University, Shanghai, China; ^3^Department of Neurology, Tongren Hospital, Shanghai Jiao Tong University School of Medicine, Shanghai, China

**Keywords:** cardioembolic stroke, ischemic stroke, biomarker, autophagy, neutrophil

## Abstract

**Introduction:**

This study aimed to investigate the shared molecular mechanisms underlying cardioembolic stroke (CS) and ischemic stroke (IS) using integrated bioinformatics analysis.

**Methods:**

Microarray datasets for the CS (GSE58294, blood samples from CS and controls) and IS (GSE16561, blood from IS and controls; GSE22255, peripheral blood mononuclear cells from IS and matched controls) were acquired from the Gene Expression Omnibus database. Differential expression analysis and weighted gene co-expression network analysis were utilized to identify shared genes between the two diseases. Protein-protein interaction (PPI) network and topology analyses were conducted to identify the core shared genes. Three machine learning algorithms were employed to detect biomarkers from the core shared genes, and the diagnostic value of the hub genes was evaluated by establishing a predictive nomogram. Immune infiltration was evaluated using single-sample gene set enrichment analysis (ssGSEA), and pathways were analyzed with gene set enrichment analysis.

**Results:**

There were 125 shared up-regulated genes and 2 shared down-regulated between CS and IS, which were mainly involved in immune inflammatory response-related biological functions. The Maximum Clique Centrality algorithm identified 25 core shared genes in the PPI network constructed using the shared genes. ABCA1, CLEC4E, and IRS2 were identified as biomarkers for both CS and IS and performed well in predicting the onset risk of CS and IS. All three biomarkers were highly expressed in both CS and IS compared to their corresponding controls. These biomarkers significantly correlated with neutrophil infiltration and autophagy activation in both CS and IS. Particularly, all three biomarkers were associated with the activation of neutrophil extracellular trap formation, but only in the IS.

**Conclusion:**

ABCA1, CLEC4E, and IRS2 were identified as potential key biomarkers and therapeutic targets for CS and IS. Autophagy and neutrophil infiltration may represent the common mechanisms linking these two diseases.

## 1 Introduction

Stroke is one of the leading causes of death worldwide, placing a heavy burden on both individuals and society, particularly as population aging has become a key feature of demographic development (1, 2). The stroke burden has increased substantially (70% and 102% increase in incidence and prevalence, and 43% increase in deaths) from 1990 to 2019, based on the Global Stroke Fact Sheet 2022 published by the World Stroke Organization (3). In China, the incidence and mortality rates of stroke increased by 86.0% and 32.3%, respectively, from 1990 to 2019 (4). Ischemic stroke (IS) is the most common type of stroke, accounting for 87% of all strokes (5). IS can be caused by an interruption of cerebral blood flow from multiple events, such as embolism of cardiac origin, occlusion of small vessels in the brain, and atherosclerosis, which initiate a series of pathophysiological processes, including immune cell infiltration and neuronal death (6). Despite current advances in medical intervention, treatment options for IS remain limited (7, 8), emphasizing the need to illustrate the mechanisms of IS and develop new therapeutic targets.

Cardiogenic cerebral embolism, also termed cardioembolic stroke (CS), refers to the clinical syndrome of cerebral artery embolism caused by cardioembolic embolism from the heart and aortic arch through circulation (9). CS is a major subtype of IS, which accounts for approximately 20%-30% of all IS cases worldwide (10, 11). Compared with IS caused by other etiologies, CS is more severe, has a worse prognosis, and has a higher recurrence rate (12). However, it is worth noting that CS has a missed diagnosis rate as high as 10%−15% (13). In addition, differences in etiology and embolus composition across different stroke subtypes determine the differences in treatment methods (9, 14). For example, patients with CS often requires oral anticoagulants to prevent recurrent events (15, 16). Therefore, illustrating the similarities and differences in the molecular expression and regulatory mechanisms between CS and other IS is of great significance in the clinical management of stroke. Nevertheless, this issue has rarely been investigated.

In this study, genes associated with CS and IS were screened independently using differential analysis and weighted gene co-expression network analysis (WGCNA), followed by screening for shared genes between these two stroke subtypes. Next, three machine learning algorithms were employed to identify core biomarkers for these two diseases. Subsequently, the associations of these biomarkers with immune infiltration and biological pathways as well as the molecular drug regulatory network for biomarkers were explored in both CS and IS. This study revealed inherent connections between the CS and IS, which may contribute to the clinical management of stroke.

## 2 Materials and methods

### 2.1 Data acquisition and preprocessing

The gene expression profiles of CS (GSE58294) and IS (GSE16561 and GSE22255) used in this analysis were downloaded from the Gene Expression Omnibus database using the R package GEOquery (version 2.66.0). Dataset GSE58294 for CS comprised of 90 blood samples from 69 CS patients and 23 normal controls. Dataset GSE16561 for IS contained 63 blood samples from 39 patients with IS and 24 healthy controls and was used as the discovery dataset. Dataset GSE22255 for IS contained 40 peripheral blood mononuclear cells samples from 20 patients with IS and 20 healthy controls; 15 IS samples and 17 control samples were retained after eliminating outlier samples. This dataset was used as the validation dataset for IS. No additional dataset for CS was retrieved from the GEO database, and therefore no external validation dataset was utilized for CS in this study. The raw microarray data were pre-processed individually for quality control (including background adjustment and normalization) by robust multi-array average (RMA). The count value was converted to log2 (cpm+1) expression data for analysis. Probes ID were converted into gene symbol based on the corresponding annotation file of the platform, and the probes matched no gene symbol were removed.

### 2.2 Differential expression analysis

Differentially expressed genes (DEGs) between the CS and control samples in the GSE58294 dataset and between the IS and control samples in the GSE16561 dataset were screened using the R package Limma (version 3.54.2), followed by Benjamini & Hochberg corrections for multiple tests. The cut-off values of |logFC| > 0.263 and adjusted *P* < 0.05 were utilized for screening of DEGs.

### 2.3 WGCNA

The R package WGCNA (version 1.72-1) was run to identify the CS- and IS-associated gene modules. The top 5,000 genes ranked by the median absolute deviation in the discovery dataset were selected for analysis. To remove outliers from the sample, hierarchical clustering analysis was conducted utilizing the “hclust” function, coupled with “method = average” as parameter for calculating distance. Next, a soft-threshold power was determined (the scale-free topological fit index R^2^ reached 0.8 for the first time) to establish an unsupervised co-expression matrix that approached a scale-free network. A gene hierarchical clustering dendrogram and dynamic tree cutting were conducted to identify highly correlated gene modules. Finally, Pearson correlations were performed to identify CS and IS-associated gene modules.

### 2.4 Shared genes between CS and IS

The DEGs of CS and IS, as well as the corresponding module genes, were intersected to obtain the shared genes across the two diseases. Gene ontology and Kyoto Encyclopedia of Genes and Genomes (KEGG) pathway enrichment analysis were performed utilizing R package clusterProfiler (version 4.6.2) to explore potential biological functions and signaling pathways associated with these shared genes, with Benjamini and Hochberg method employed for multiple-testing correction. The adjusted *P* < 0.05 and count ≥ 2 was utilized as cut-off values. Protein–protein interactions (PPI) among these shared genes were predicted utilizing the Search Tool for the Retrieval of Interacting Genes/Proteins (STRING) database, and the PPI network was visualized using Cytoscape software (version 3.10.2). The Maximum Clique Centrality (MCC) method of the CytoHubba plug-in was applied to screen the top 25 genes in the PPI network.

### 2.5 Machine learning for identifying diagnostic biomarkers

Three machine learning algorithms, lasso-logistic, Boruta, and Support Vector Machine-Recursive Feature Elimination (SVM-REF), were employed to select potential diagnostic biomarkers from shared genes. Specifically, lasso-logistic analysis was conducted utilizing the R package glmnet (version 4.1-8) with 5-fold cross-validation, while Boruta analysis was conducted using the R packages Boruta (version 8.0.0). SVM-RFE is a feature selection method based on SVM, which was carried out with 10-fold cross validation by using R package e1071 (version 1.7-14). The feature genes identified by each algorithm were merged to obtain candidate diagnostic biomarkers. The expression of these candidate biomarkers in both discovery and validation datasets was analyzed. The predictive power of these candidate biomarkers was assessed by plotting receiver operating characteristic (ROC) and precision-recall (PR) curves. Only those with consistent differential expression in both discovery and validation datasets and an area under the ROC curve (AUROC) and PR curve (AUPRC) over 0.6 were finally selected as biomarkers. To facilitate the clinical use of these identified biomarkers, a predictive Nomogram was established using the R package “rms” (version 6.7-1). The accuracy and clinical value of the Nomogram model was further evaluated through calibration curve and decision curve analysis, which were plotted utilizing the calibrate method provided in “rms” package and the R package rmda (version 1.6), respectively.

### 2.6 Evaluation of immune infiltration

The infiltration fractions of 28 types of immune cells in tissue samples were inferred using single-sample gene set enrichment analysis (ssGSEA), which was conducted through the R package GSVA (version 1.46.0). In addition, differences in the infiltration fractions of each immune cell type across the disease and control groups were assessed using *t-*tests (*P* < 0.05). Pearson's correlation analysis was performed to determine the relationship between biomarkers and infiltrating immune cells.

### 2.7 Construction of regulatory networks

The interacting genes and their functions in the identified biomarkers were further analyzed using the GeneMANIA database (http://genemania.org/). Transcription factors and microRNA (miRNAs) that may target biomarkers were predicted utilizing the online tool NetworkAnalyst.

### 2.8 Small molecule drug prediction and molecular docking

Small molecule drugs that may target biomarkers were predicted using the dgidb database. To gain insight into how the drugs bind to key genes, we performed a molecular docking analysis. Briefly, the three-dimensional (3D) structures of the drugs were acquired from the PubChem database, and the protein structures corresponding to the biomarkers were predicted using the R package AlphaFold (version 2.0). Subsequently, CB-Dock (version 1.0) was employed to simulate molecular docking, and the results were visualized using the PyMOL software (version 3.0).

### 2.9 Gene set enrichment analysis

To illustrate the biological functions of biomarkers, disease samples were categorized into high- and low-expression groups based on the median value, and the deregulated pathways across the expression groups were explored through GSEA. Briefly, with the KEGG gene set as an enrichment reference, GSEA analysis was performed utilizing the R package clusterProfiler, and the threshold values were adjusted to *P* < 0.05 and |normalized enrichment Score (NES)| > 1.

## 3 Results

### 3.1 Screening of key dysregulated genes in CS and IS

**Figure 1:**
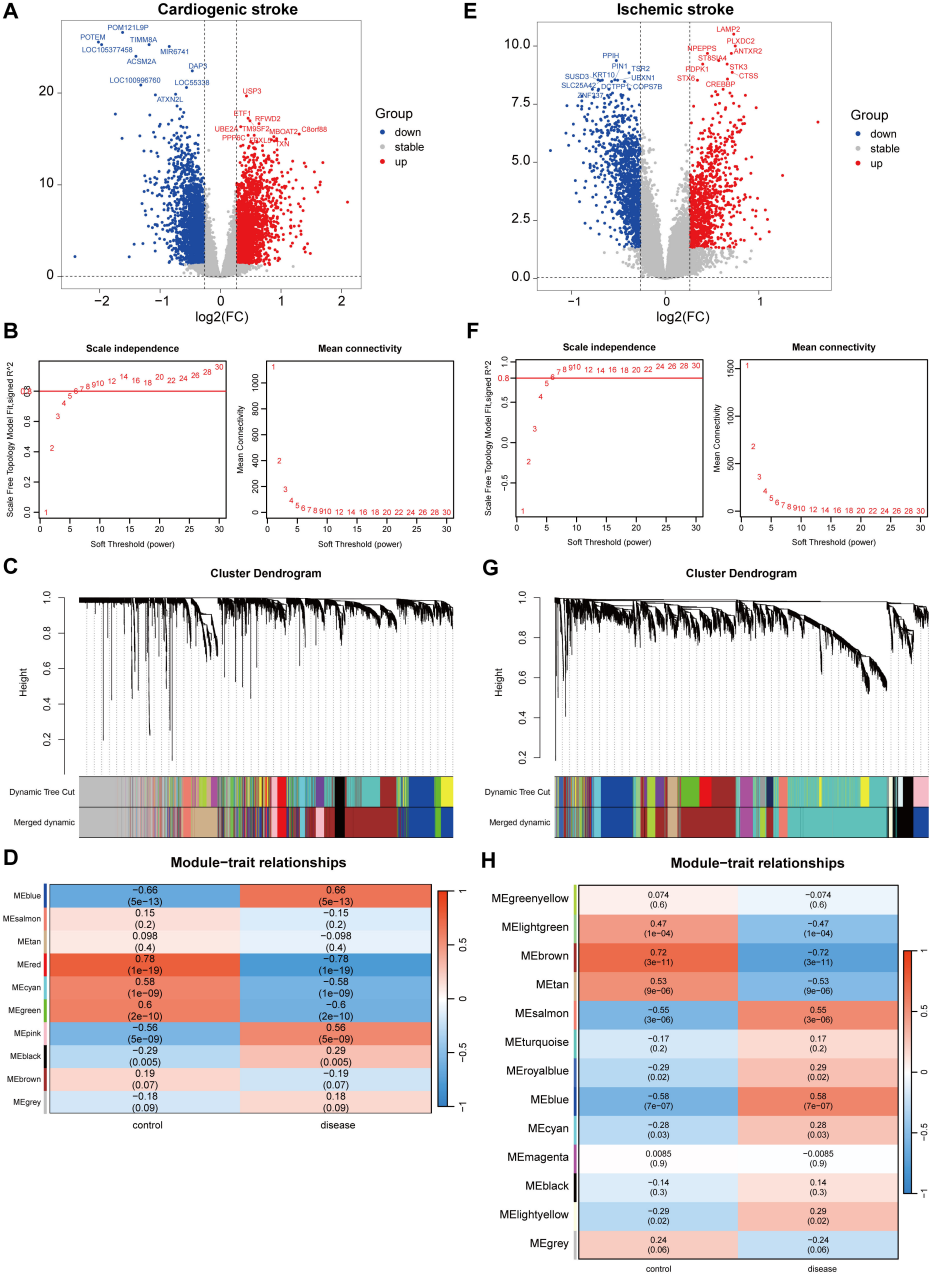
Identification of gene modules associated with cardioembolic stroke (CS) and ischemic stroke (IS). **(A, E)** Volcano plots showing the differentially expressed genes (DEGs) between CS and control samples or between IS and control samples. The red and blue dots refer to the up-regulated and down-regulated genes, and the gray dots refer the genes with no significant changes on their expression; **(B, F)** calculation of soft threshold (power) in weighted gene co-expression network analysis (WGCNA); **(C, G)** cluster dendrogram generated through hierarchical clustering based on dissimilarity measures of genes; **(D, H)** heatmaps of module-trait relationships.

In the GSE58294 dataset, there were 4,591 DEGs between the CS and control samples. Of which, the expression of 2,272 genes increased, whereas the expression of 2,319 genes decreased in the CS samples (Figure 1A). Gene modules highly associated with CS were further screened utilizing WGCNA, and a soft-threshold power of 10 was selected to balance the relationship between mean connectivity and scale independence (Figure 1B). A total of 15 gene modules were identified, with a minimum of 50 genes per gene module, and 10 modules were determined when merging the modules with 75% correlation (Figure 1C). Heatmap of module–trait relationships showed that “blue” module was positively correlated with CS (r = 0.66, *P* = 5e-13, Figure 1D). Therefore, the 817 genes in this “blue” module were regarded as CS-associated module genes.

In the GSE16561 dataset, there were 2,473 DEGs between the IS and control samples, including 1,069 upregulated and 1,404 downregulated genes (Figure 1E). WGCNA was conducted to identify gene modules highly associated with IS, and a soft threshold power of 7 was selected (Figure 1F). A total of 21 gene modules were identified, with the minimum number of genes per gene module set to 50, and 13 modules were determined when merging the modules with 75% correlation (Figure 1G). Among the 13 modules, “blue” module was positively correlated with IS (r=0.58, *P* = 7e-07, Figure 1H), and 673 genes in this module were obtained.

### 3.2 Shared hub genes in CS and IS

**Figure 2:**
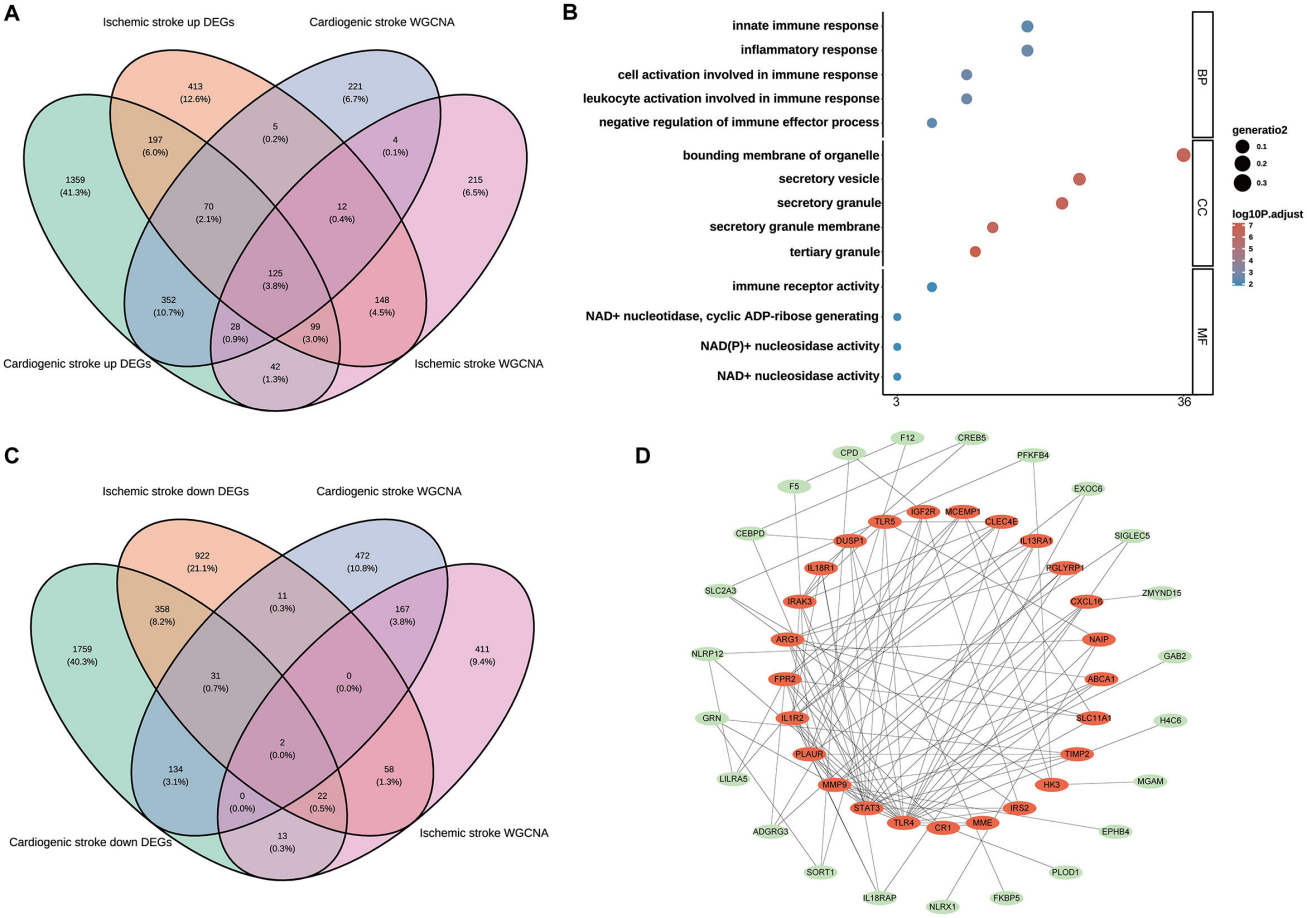
Identification of the shared genes in cardioembolic stroke (CS) and ischemic stroke (IS). **(A)** Venn diagram showing the shared upregulated genes between CS and IS; **(B)** the top five terms of three gene ontology categorizations; **(C)** Venn diagram depicting the shared downregulated genes between CS and IS; **(D)** the PPI network of the shared genes, in which the red nodes represent the top 25 hub genes identified from the PPI network using the Maximum Clique Centrality (MCC) method.

Among the upregulated DEGs for CS (*n* = 2,272) and IS (*n* = 1,069), as well as the module genes for CS (*n* = 817) and IS (*n* = 673), 125 shared genes were screened (Figure 2A). These genes were significantly enriched in biological processes related to the immune inflammatory response, such as leukocyte activation, negative regulation of immune effector processes, and inflammatory responses. Consistently, these genes were also markedly enriched in the molecular function terms of immune receptor activity (Figure 2B), indicating their involvement in immune inflammation-related functions. Only KEGG pathway of inflammatory bowel disease was enriched with the cut-off values of adjusted *P* < 0.05 and count ≥2 (Supplementary Table 1). Two shared genes were further screened from the downregulated DEGs for CS (*n* = 2,319) and IS (*n* = 1,404), as well as the module genes for CS (*n* = 817) and IS (*n* = 673), as shown in Figure 2C. The enrichment results of these two genes (ZNF83 and THOC1) are displayed in Supplementary Table 1. However, no significant enrichment terms were determined under the cut-off values of adjusted *P* < 0.05 and count ≥2 owing to the limited gene number. Interestingly, we found that THOC1 was enriched in multiple immune-related terms such as negative regulation of immunoglobulin-mediated immune response, negative regulation of B cell activation, and negative regulation of lymphocyte-mediated immunity (Supplementary Table 1). We further investigated the interactions between 127 shared genes and constructed a PPI network (Figure 2D). From this network, the top 25 genes were determined using the MCC algorithm, and close interactions were observed among these 25 hub genes (Figure 2D). These 25 genes were selected for subsequent analysis.

### 3.3 Determination of candidate biomarkers through machine learning

**Figure 3:**
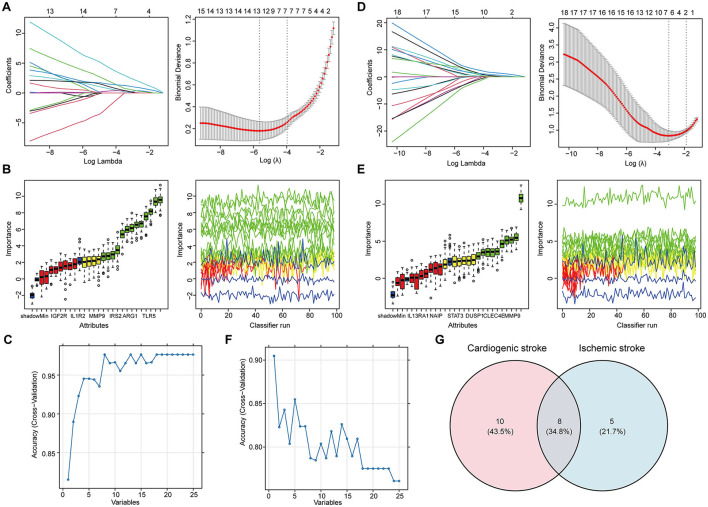
Determination of the shared feature genes in cardioembolic stroke (CS) and ischemic stroke (IS). **(A, D)** Parameter selection for the LASSO regression. (left) Cross-validation to select the optimal parameter lambda, and (right) distribution of the LASSO coefficient for feature genes; **(B, E)** results of the Boruta algorithm for identifying feature genes by assessing their importance; **(C, F)** results of SVM-RFE algorithm for screening feature genes with the highest accuracy, in which the horizontal coordinate refers to the number of genes, and the vertical coordinate refers to the accuracy under 10-fold cross-validation; **(G)** Venn diagram showing the eight shared feature genes between CS and IS.

Feature selection from the 25 shared hub genes was conducted using three machine learning algorithms. In the context of CS, LASSO logistic regression (Figure 3A) and Boruta analysis (Figure 3B) each identified 13 feature genes. SVM-RFE identified eight feature genes with the highest accuracy of 0.976 in 10-fold cross-validation (Figure 3C). In total, 18 genes that were considered candidate biomarkers in CS were obtained through these three algorithms after removing redundancies (Supplementary Table 2).

For feature gene screening in the context of IS, LASSO regression determined seven genes (Figure 3D), and Boruta analysis identified 10 genes (Figure 3E). Among the 25 hub genes, only one was identified as a key feature gene for IS using the SVM-RFE algorithm, with the highest accuracy of 0.9 (Figure 3F). Following the union of the genes obtained from the three algorithms, 13 feature genes were determined in the IS (Supplementary Table 2). Ultimately, eight candidate biomarkers shared between CS and IS were screened: IGF2R, IRAK3, TLR4, ABCA1, CXCL16, CLEC4E, ARG1, and IRS2 (Figure 3G).

### 3.4 Determination of diagnostic biomarkers by assessing expression and predictive performance

**Figure 4:**
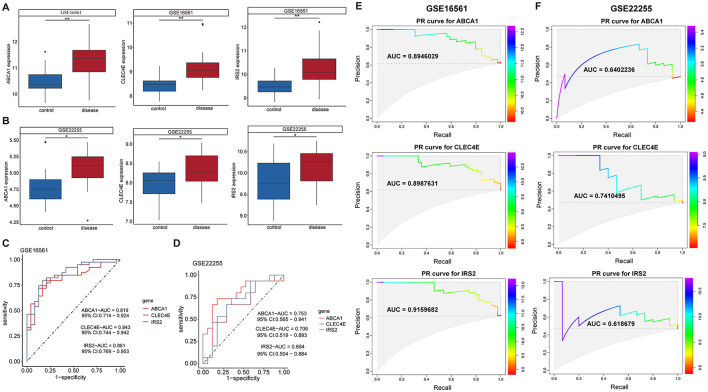
Expression and predictive performance of diagnostic biomarkers in ischemic stroke (IS). Expression of the three biomarkers in training set GSE16561 **(A)** and validation set GSE22255 **(B)**; Receiver operating characteristic (ROC) curves showing the predictive power of the three biomarkers for IS in training set GSE16561 **(C)** and validation set GSE22255 **(D)**. Precision-recall (PR) curves showing the predictive power of the three biomarkers for IS in training set GSE16561 **(E)** and validation set GSE22255 **(F)**. ^*^P < 0.05; ^**^P < 0.01.

Further screening of the eight candidate biomarkers was performed to identify additional weighted diagnostic biomarkers. As described above, only those with consistent differential expression in both the discovery and validation datasets and an AUC over 0.6 were finally selected. This screening step was conducted in the IS but not in the CS because there was only one CS dataset. In both the training set GSE16561 and validation set GSE22255, the expression of ABCA1, CLEC4E, and IRS2 was elevated in IS samples compared to that in normal controls (Figures 4A, B). In addition, these three genes performed well in distinguishing IS samples, with AUROC of 0.819, 0.843, and 0.861 for ABCA1, CLEC4E, and IRS2, respectively, in the training set GSE16561 (Figure 4C). Similarly, in the validation set GSE22255, the AUROC for ABCA1, CLEC4E, and IRS2 were 0.753, 0.706, and 0.694 (Figure 4D), respectively, indicating moderate predictive power for IS. The predictive performance of these three genes were also assessed by PR curves. In the training set GSE16561, the AUPRC for ABCA1, CLEC4E, and IRS2 were 0.895, 0.899, and 0.916, respectively (Figure 4E). In the validation set GSE22255, the AUPRC for ABCA1, CLEC4E, and IRS2 were 0.640, 0.741, and 0.619 (Figure 4F). The AUROC and AUPRC were all over 0.6 for these three genes in both training and validation sets. Therefore, ABCA1, CLEC4E, and IRS2 were identified as potential diagnostic biomarkers.

### 3.5 Construction of clinical predictive nomogram for CS and IS

**Figure 5:**
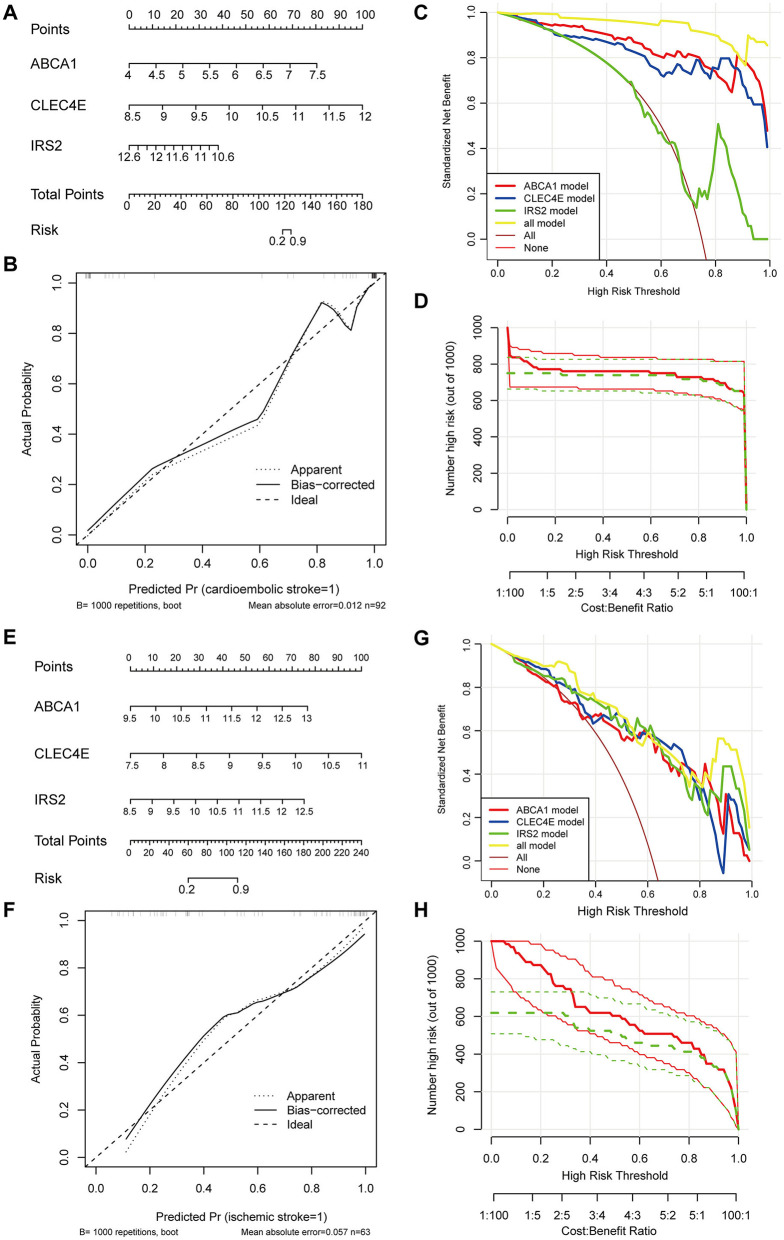
Establishment and evaluation of predictive Nomogram. Nomogram established using three biomarkers for predicting the onset risk of cardioembolic stroke (CS) **(A)** and ischemic stroke (IS) **(E)**; calibration curve for assessing the accuracy of nomogram in predicting CS **(B)** and IS **(F)**; decision curve for evaluating the clinical benefit of nomogram in CS **(C)** and IS **(G)**; clinical impact curve for evaluating the clinical benefit of nomogram in CS **(D)** and IS **(H)**.

To facilitate the clinical use of the identified biomarkers, a predictive nomogram was established for CS and IS based on the three identified biomarkers, ABCA1, CLEC4E, and IRS2 (Figures 5A, B). In the Nomogram for both CS and IS, CLEC4E harbored the highest weight among the three genes (Figures 5A, B). The high conformance of the predicted dotted line with the actual calibration curve suggested that the nomogram had outstanding accuracy in predicting the onset risk of CS and IS (Figures 5C, D). The greatest net benefit of the model with all three genes compared with that with a single characteristic gene in the decision curve further demonstrated the high accuracy of the predictive nomogram in predicting the risk of CS and IS (Figures 5E, F). The clinical impact curve further confirmed the conformance between the predicted and actual probabilities in CS and IS (Figures 5G, H), implying the clinical applicability of the nomogram.

### 3.6 Biomarkers expression correlated with immune cell abundance in CS and IS

**Figure 6:**
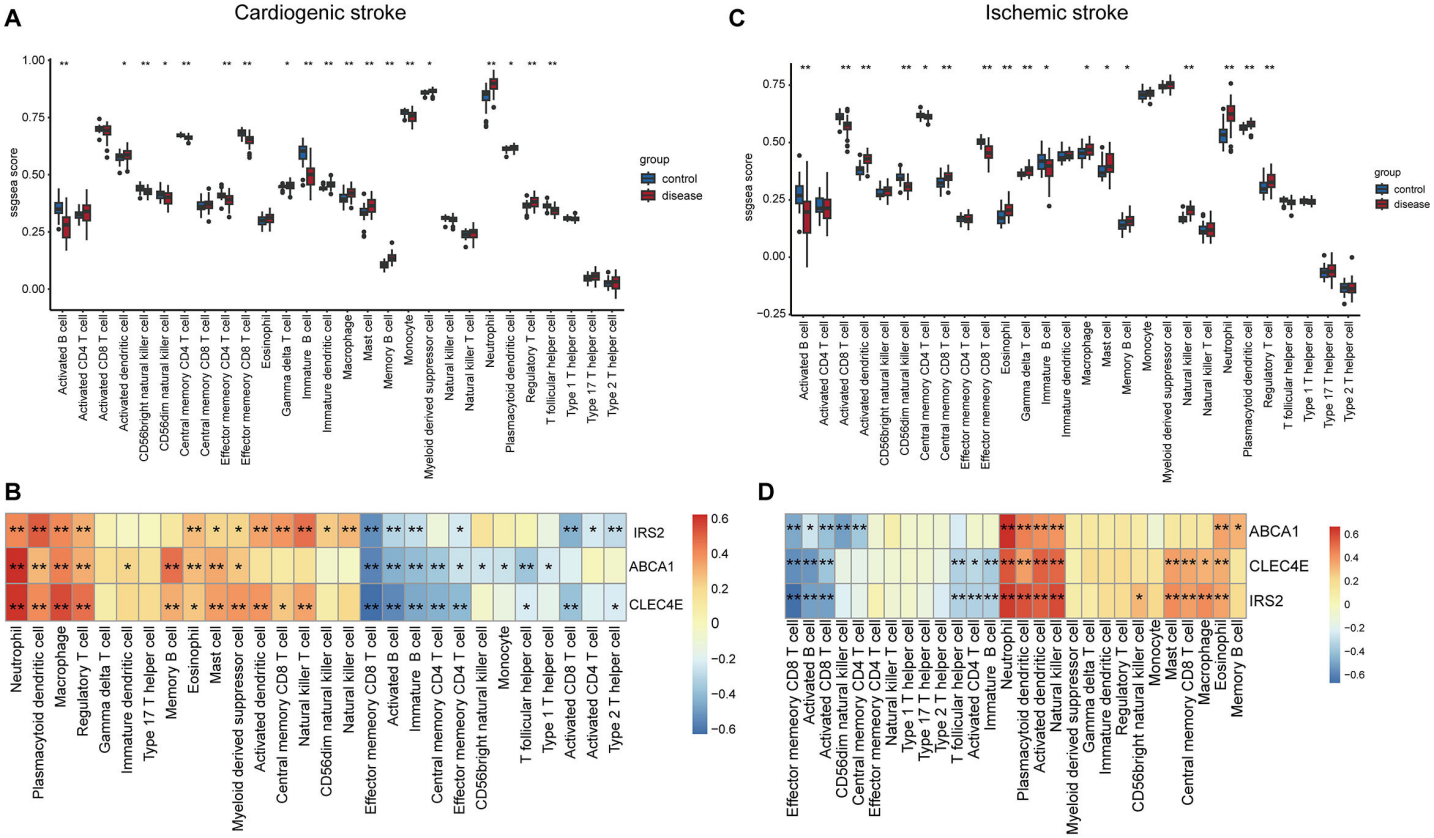
Correlations of biomarkers with immune cells. Boxplots showing the differences in the infiltration levels of 28 immune cells between disease and control samples in cardioembolic stroke (CS) **(A)** and ischemic stroke (IS) **(C)**. The comparisons between disease and control groups were conducted using *t*-tests; correlation heatmaps revealing the relationships between the three biomarkers and the immune cells in CS **(B)** and IS **(D)**. ^*^P < 0.05; ^**^P < 0.01.

Immune cells in the samples were inferred using ssGSEA based on gene expression profiles. In the CS samples, there were 19 immune cells with an abundance markedly different from that in the normal controls (Figure 6A). For instance, CS samples harbored a lower abundance of activated/immature B cells and effector memory CD4+/CD8+ T cells and a higher abundance of macrophages, mast cells, and neutrophils (Figure 6A). The correlations between biomarker expression and immune cell abundance were analyzed. All three biomarkers positively correlated with the levels of multiple cells, such as neutrophils, macrophages, and regulatory T cells (Tregs) and negatively correlated with cells including activated/immature B cells and effector memory CD4+/CD8+ T cells (Figure 6B, Supplementary Figure 1A).

In the context of IS, there were 17 immune cells, with their abundance markedly differing between IS and normal controls (Figure 6C). Consistently, the IS samples also exhibited a lower abundance of activated/immature B cells and effector memory CD8+ T cells and a higher abundance of macrophages, mast cells, and neutrophils (Figure 6C). Correlation analysis suggested that the three biomarkers were positively correlated with the levels of neutrophils, plasmacytoid/activated dendritic cells, and natural killer cells and negatively correlated with effector memory CD8+ T cells, activated B cells, and activated CD8+ T cells (Figure 6D, Supplementary Figure 1B).

### 3.7 Biomarker-associated pathways in CS and IS

**Figure 7:**
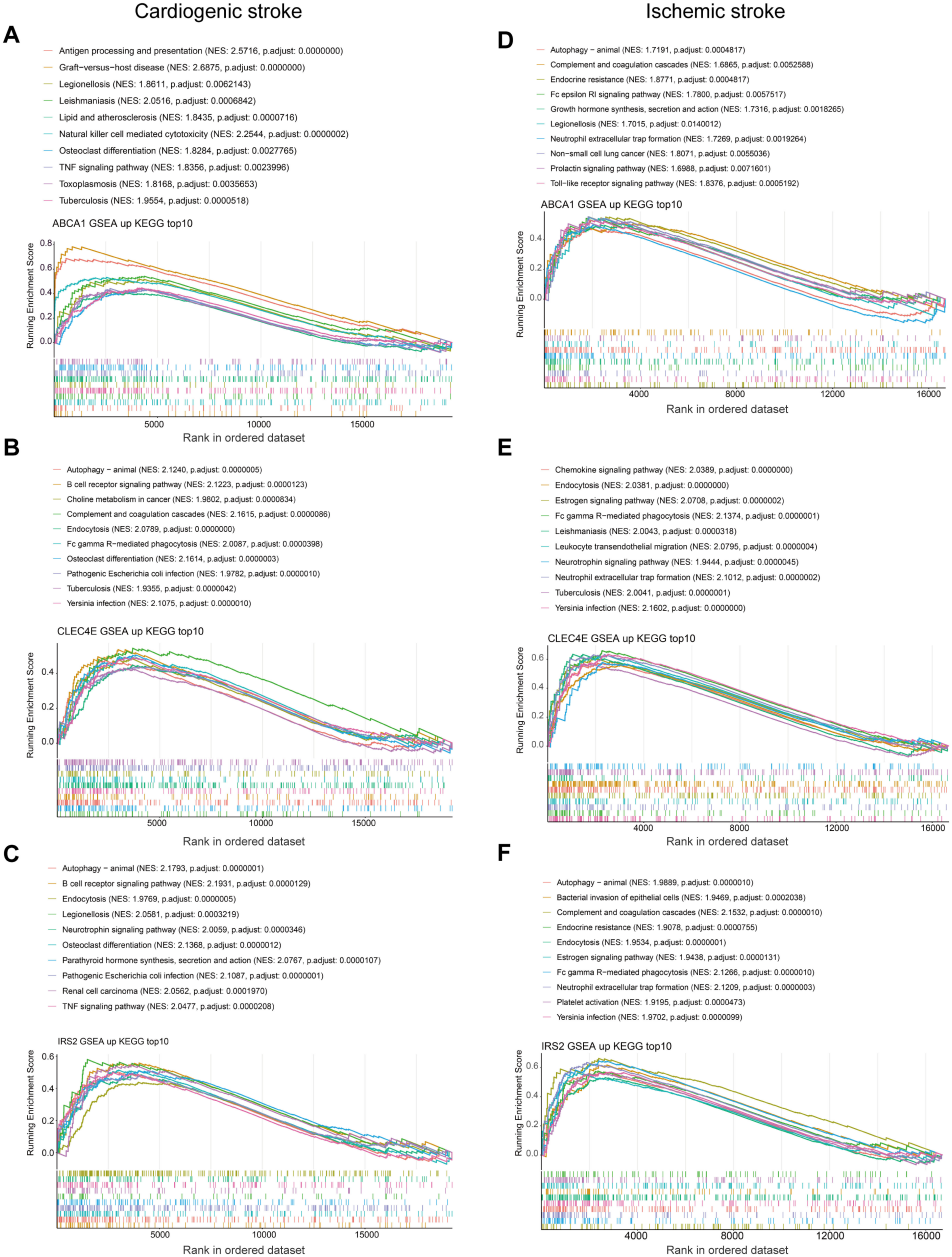
Gene set enrichment analysis. Top 10 activated KEGG pathways with ABCA1 expression in cardioembolic stroke (CS) **(A)** and ischemic stroke (IS) **(D)**; top 10 activated KEGG pathways with CLEC4E expression in CS **(B)** and IS **(E)**; top 10 activated KEGG pathways with IRS2 expression in CS **(C)** and IS **(F)**.

To discover the KEGG pathways probably affected by biomarkers expression, we performed GSEA for each biomarker in both diseases. In the context of CS, pathways such as antigen processing and presentation, NK cell-mediated cytotoxicity, lipids, and atherosclerosis were activated, with increased ABCA1 expression (Figure 7A). Elevated expression of CLEC4E and IRS2 was activated through the activation of autophagy and the B-cell receptor signaling pathway (Figures 7B, C). However, multiple pathways related to metabolism and nucleotide excision repair were inhibited (Supplementary Figures 2A–C). Interestingly, the elevated expression of biomarkers was accompanied by the activation of autophagy in the IS. In addition, neutrophil extracellular trap (NET) formation was observed (Figures 7D–F). Ribosome biogenesis-related pathways were inhibited with the expression of these three biomarkers (Supplementary Figures 2D–F). Overall, autophagy was a common pathway activated in both CS and IS.

### 3.8 Regulatory networks for biomarkers

**Figure 8:**
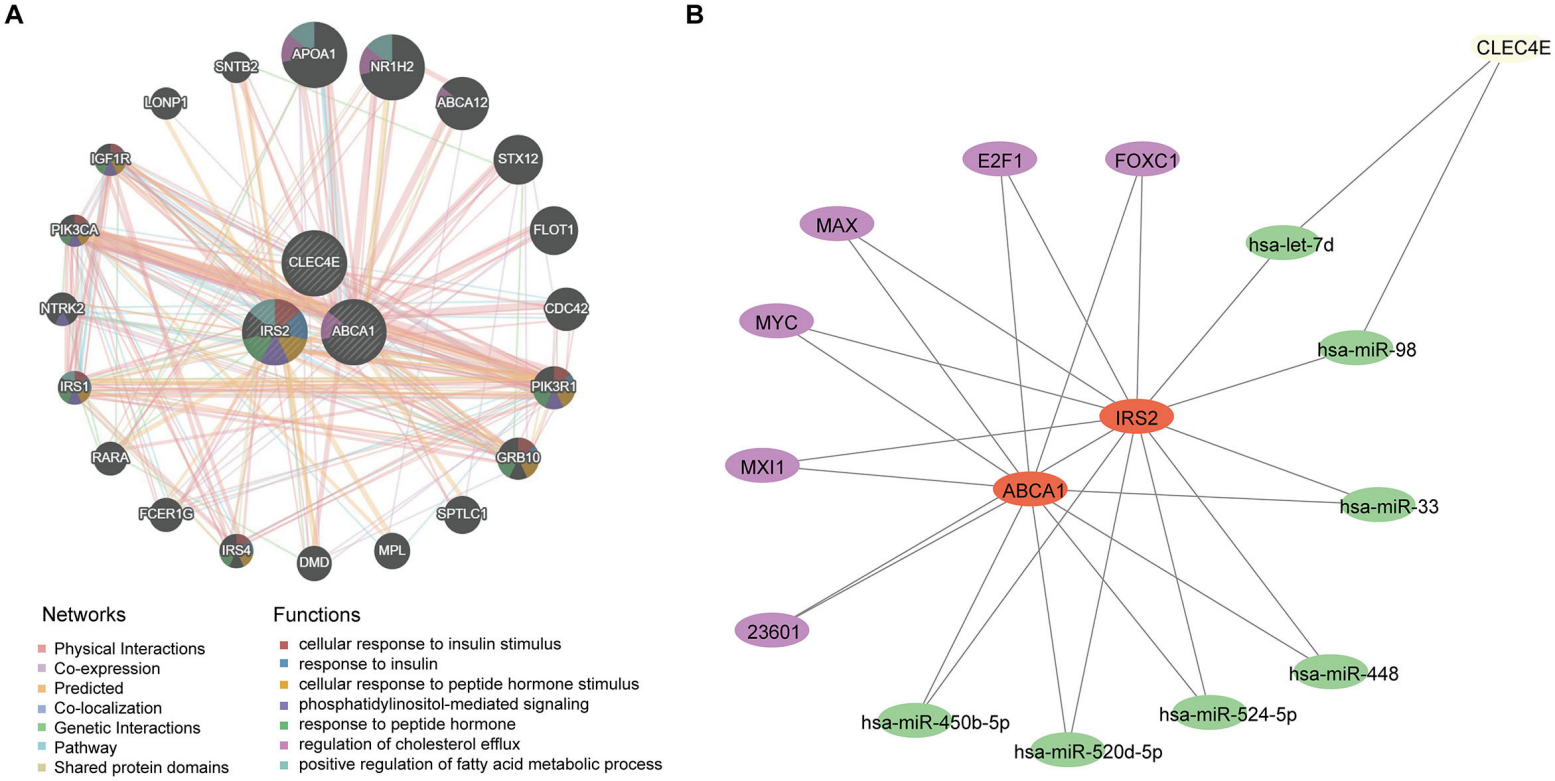
Regulatory network for biomarkers. **(A)** Interaction network and involved functions of ABCA1, CLE4E, and IRS2 using GeneMANIA; **(B)** microRNAs and transcription factors for ABCA1, CLE4E, and IRS2, in which the purple nodes represent transcription factors and the green nodes indicate miRNAs.

**Figure 9:**
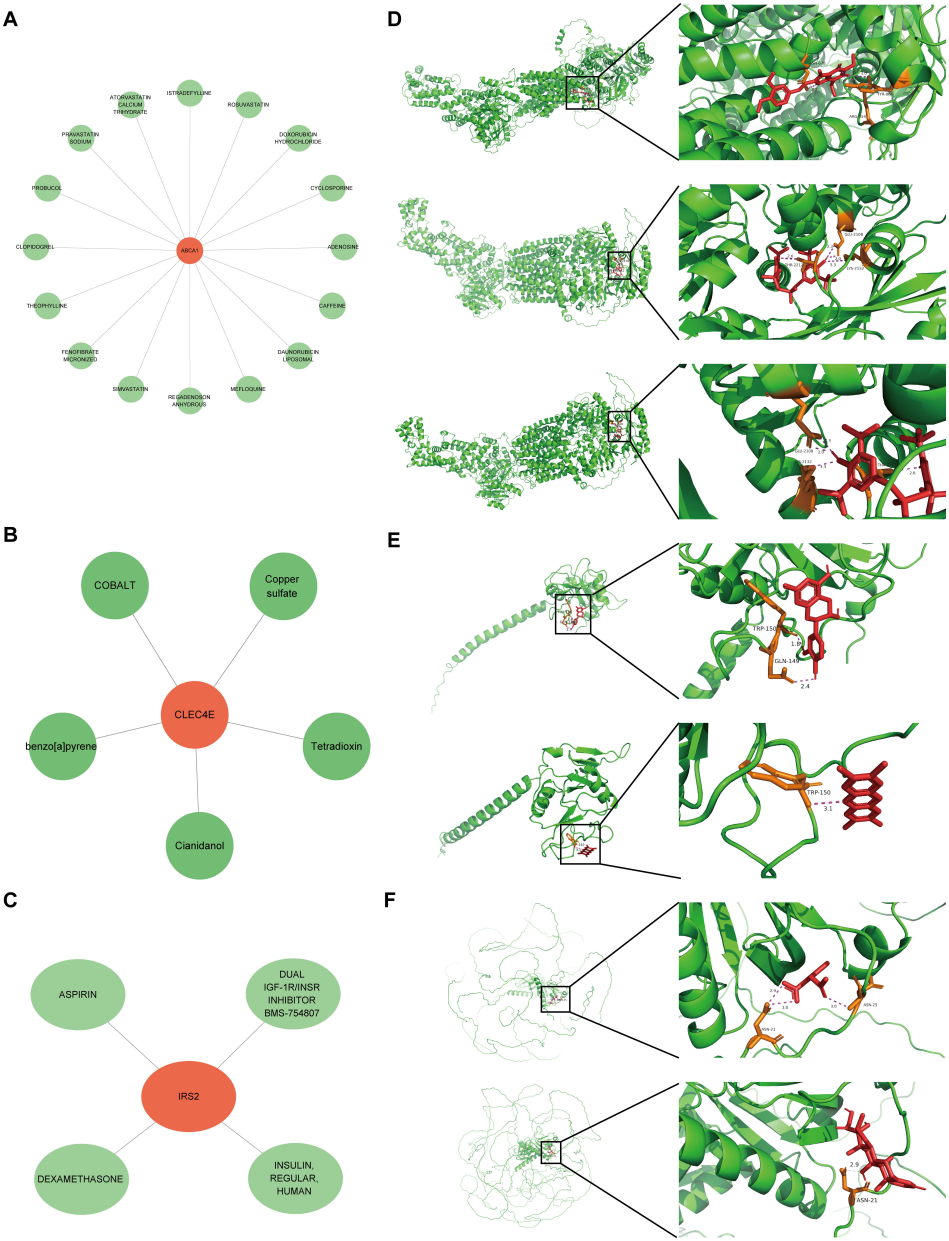
Small molecular drugs for targeting biomarkers. Small molecular drug network for ABCA1 **(A)**, CLE4E **(B)** and IRS2 **(C)**, in which the green nodes refer to the drugs; **(D)** results of molecular docking for ABCA1 and the corresponding top three drugs probucol, mefloquine, and istradefylline; **(E)** results of molecular docking for CLEC4E and drugs Cianidanol and Tetradioxin; **(F)** results of molecular docking for IRS2 and drugs aspirin and dexamethasone.

A potential molecular regulatory mechanism was identified to provide a comprehensive understanding of the three biomarkers. GeneMANIA analysis revealed that ABCA1, CLEC4E, IRS2, and their interacting genes were mainly involved in the cellular response to insulin stimulus, cellular response to peptide hormones, and regulation of cholesterol efflux (Figure 8A). Regarding molecular regulation, both ABCA1 and IRS2 were likely targeted by multiple miRNAs and transcription factors (Figure 8B), indicating the potential of these two genes as therapeutic targets. Therefore, we predicted the drugs that could target these three genes. Sixteen drugs were predicted for ABCA1, while four and five drugs were predicted for IRS2 and CLEC4E, respectively (Figures 9A–C). Molecular docking was conducted to confirm the binding of the genes to predicted representative drug molecules. For ABCA1, docking was conducted for ABCA1 and the top three drugs: probucol, mefloquine, and istradefylline. Five docking models were obtained, and the model with the lowest binding energy (best affinity) was selected. The binding energy of ABCA1 with probucol, mefloquine, and istradefylline were −8.8, −8.8, and −7.5 kcal/mol, respectively (Figure 9D, Supplementary Table 3). For the five drugs predicted for IRS2, docking was only conducted for aspirin and dexamethasone (Figure 9E) because of the unavailability of 3D structures of the other three drugs. The binding energy of IRS2 with aspirin and dexamethasone were −5.4 and −6.7 kcal/mol (Supplementary Table 3). Similarly, docking was conducted for CLEC4E with Cianidanol and Tetradioxin (Figure 9F), and the binding energy was −6.4 and −6.0 kcal/mol, respectively (Supplementary Table 3). The docking results confirmed the binding of these genes to the predicted drugs with high affinity.

## 4 Discussion

CS is the major IS subtype. The etiology and pathogenesis of different stroke subtypes are diverse, leading to variations in their treatment. Therefore, illustrating the similarities and differences in the molecular mechanisms of different stroke subtypes can contribute to an accurate early diagnosis and a more targeted therapeutic schedule for patients with stroke. In this study, we revealed the overlapping molecular mechanisms across the two stroke subtypes through integrated bioinformatics analyses.

Based on differential analysis and WGCNA, we identified 127 shared differential genes between CS and IS. These genes were mainly implicated in biological processes related to immune inflammatory responses, such as leukocyte activation and negative regulation of immune effector processes. Immune-inflammatory response exerts vital and bidirectional roles in the pathological process of IS (17, 18). Immune cell infiltration is the core mechanism involved in the modulation of nerve injury and repair after stroke (19). In stroke brain tissue, some types of infiltrated T cells promote inflammatory responses to aggravate tissue injury, while T cells contribute to protecting neurons from ischemic injury by inducing immunosuppression (20–23). Currently, immunological mechanisms are a hotspot of research in the field of IS, and targeting the immune-inflammatory response has been proposed as a promising therapeutic strategy to improve the injury post stroke (18, 23). A previous study demonstrated that FOXP3+ macrophages are beneficial for stroke outcomes by inhibiting IS-induced neural inflammation (24). Therefore, exploring alterations in the immune status of IS may provide novel insights into its management and treatment.

Machine learning is a burgeoning field in medicine that provides superior predictive power in comparison with conventional statistical models, capturing non-linear relations across predictive factors and outcomes and complex interactions within predictive factors (25, 26). Given their high accuracy, machine learning approaches are increasingly being applied in the medical field, particularly in stroke (27, 28). In this study, three machine-learning algorithms, LASSO-logistic, Boruta, and SVM-RFE, were employed to identify more weighted feature genes from shared genes. Eight feature genes were identified, which were considered candidate biomarkers for the two diseases. Further expression and predictive power assessments determined three diagnostic biomarkers—ABCA1, CLEC4E, and IRS2.

Cholesterol plays important structural and functional roles in both the gray and white matter. ABCA1, an ATP-binding cassette transporter A1, is a major membrane transporter that functions as a cholesterol efflux pump to mediate cholesterol homeostasis in the brain, particularly the efflux of cholesterol from astrocytes (29, 30). Excessive cholesterol causes fat to build up in the arteries, forming atherosclerosis and increasing the risk of cerebrovascular disease, one of the main causes of stroke (31, 32). Besides, ABCA1 modulates a variety of brain functions, such as neuroinflammation (a crucial process following stroke) and blood-brain barrier leakage, and both these two are key factors to worsen stroke outcomes (30, 33). Genetic variants of ABCA1 have been implicated in etiology and the onset risk of IS (34, 35). ABCA1 expression is implicated in the neurorestoration post stroke. For instance, specific deletion of brain-ABCA1 could reduce the density of white matter and gray matter in the ischemic brain and harm post stroke functional outcomes (29). Upregulation of ABCA1 is involved in the effects of LXR agonists in decreasing neuroinflammation, facilitating neuroprotection, and improving neurological functional-outcomes post stroke (36, 37).

CLEC4E encodes a member of the C-type lectin superfamily, which modulates immune and inflammatory responses, as well as cell-to-cell adhesion (38, 39). Although CLEC4E has not been reported in patients with stroke, other members of this superfamily have been shown to play important roles. For example, CLEC14A deficiency can exacerbate the neuronal loss post stroke by enhancing the pro-inflammatory response and blood-brain barrier permeability (40). Particularly, C-Type lectin receptor 2 has been recognized as a biomarker of platelet activation and is associated with pathological features and prognosis of strokes (41, 42). IRS2 encodes insulin receptor substrate (IRS) 2; ISR signaling mediates cardiac energy metabolism and heart failure (43) and is associated with CS (44). Gene polymorphism of IRS1 has been proposed as a risk factor for IS (45). IRS proteins are key molecular that regulates insulin signaling pathways and is strongly associated with the development of diabetes (46), while diabetes has been shown to be a risk factor for a significantly increased risk of stroke (47, 48). Nevertheless, the exact role of IRS2 in strokes remains unclear. We found that IRS2 and CLEC14A were overexpressed in both CS and IS and that their expression was associated with the risk of disease onset.

Neutrophil targeting has been proposed as a promising strategy for IS therapy (49–51). Specifically, there was a rapid increase of neutrophils in peripheral blood and in the peri-infarct cortex during all stages of IS, with enhanced neutrophil frequency linked to poor clinical outcomes (50, 52). NETs induce thrombosis by activating the clotting pathway and endothelium by acting as a scaffold for tissue factors and platelets, resulting in a procoagulant state (53). In addition, NETs released by neutrophils can mediate cerebral injury after IS. For instance, NETs facilitate thrombus formation (54) and repress vascular remodeling post-IS (52). Treatment with NET-inhibitory factors reduce cerebral infarcts and improve overall outcomes in a stroke mouse model (51). In this study, we found that all three biomarkers, ABCA1, CLEC4E, and IRS2, were associated with the activation of NET formation and infiltration levels of neutrophils in the IS, implying their importance in stroke. Autophagy was found to be a shared pathway associated with biomarkers of both diseases. Autophagy is an adaptive mechanism of the cell response to stroke and plays a vital role in maintaining cell homeostasis and survival by clearing damaged cell components via autophagic lysosomal degradation. During IS, the lack of oxygen and glucose supply caused by cerebral ischemia leads to activation of the AMPK pathway, activating autophagy in various cell types in the brain (55). Autophagy appears to play a “double-edged sword” role in the pathogenesis of IS, and its exact role in IS remains controversial, despite extensive study (56, 57). These findings further highlight the close involvement of the three identified biomarkers in stroke.

Despite the above findings, several limitations in this study should be admitted. First at all, since there was only one CS dataset, the determination of diagnostic biomarkers by assessing expression and predictive performance was conducted based solely on the IS datasets. The sample size of the dataset analyzed in this study is not large enough, which may reduce statistical power and generalizability, thus leading to certain unrobustness of the results. Second, we observed an association between the expression of three biomarkers and the activity of NET pathway, but this association appears to be observed only in IS. Such differences might be explained by the differential expression pattern of genes in the context of these two strokes. In future, the NET levels in serum/plasma samples should be tested in large number of patients to further discover whether there are differences on NETs levels between CS and IS. Besides, the causal relationship of the dysregulated status of biomarkers and NET activity should be investigated by functional experiments. Third, functional experiments are required to further confirm exact role of these three genes in stroke, mainly the similarities and differences of the actions of these three genes in the CS and IS. The last one, the drug molecules that may target these tree key genes were predicted, and the binding of the genes to predicted representative drug molecules were confirmed by molecular docking. In future, binding assays are required to confirm such drug-target interactions, and the potential applications of these drugs in strokes need to be further explored.

In summary, the current study discovered the similarities and differences in gene expression and molecular mechanisms between the two stroke subtypes to illustrate their associations. ABCA1, CLEC4E, and IRS2 were identified as common diagnostic biomarkers of both CS and IS, and their expression was associated with neutrophil infiltration and autophagy activation.

## Data Availability

The original contributions presented in the study are included in the article/Supplementary material, further inquiries can be directed to the corresponding author.
