# Supplementary material for: Exploration of the shared gene signatures and molecular mechanisms between cardioembolic stroke and ischemic stroke
Source: Front Neurol. 2025 Apr 8;16:1567902. doi: 10.3389/fneur.2025.1567902 (PMC12011848; doi:10.3389/fneur.2025.1567902)
Supplement: Supplementary file 1 [file Supplementary_file_1.docx]

Supplementary Material

# Supplementary Figures


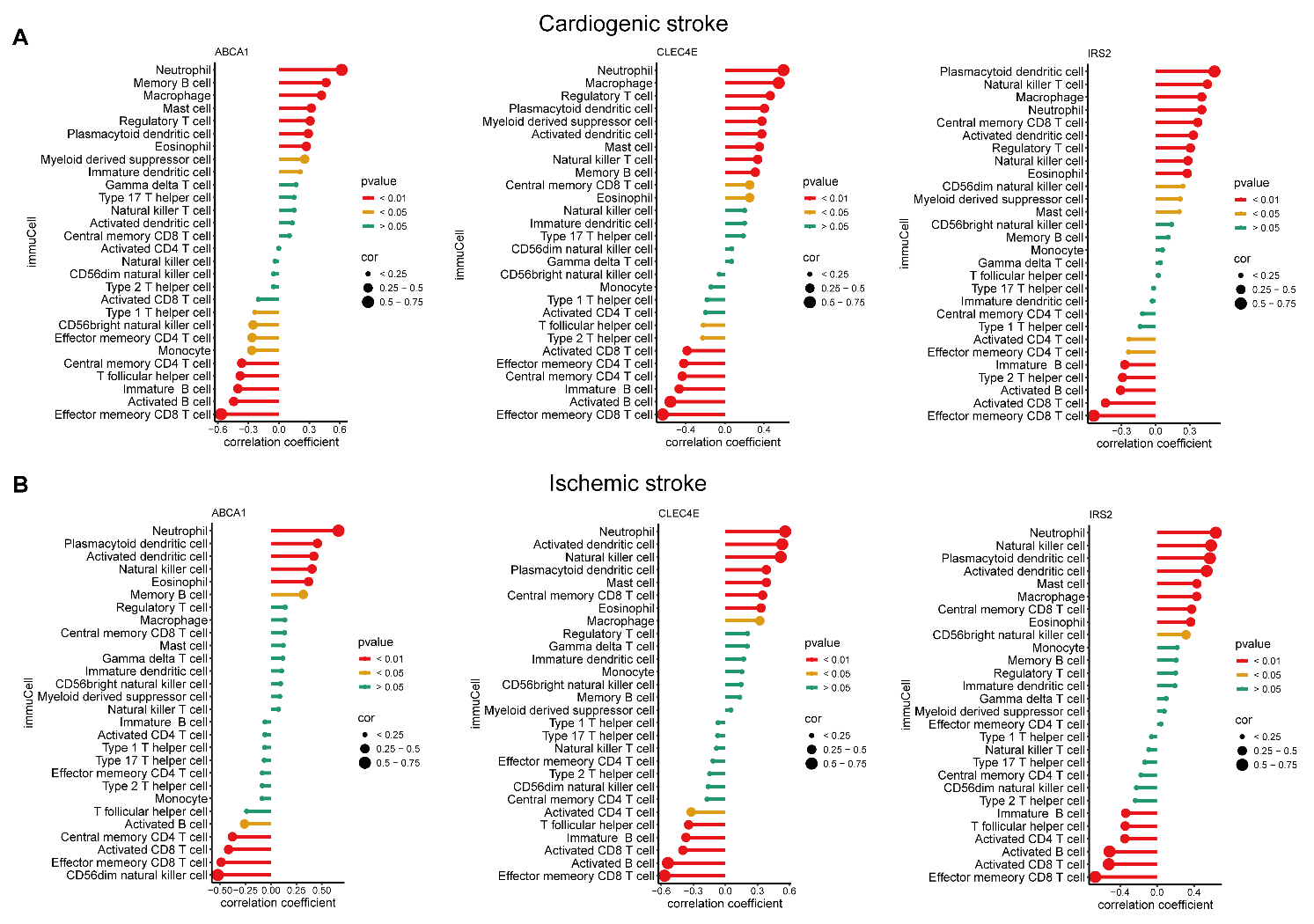


**Supplementary Figure 1. Correlations of biomarkers with immune cells.**

Lollipop diagram showing the relationships between three biomarkers and the immune cells in cardioembolic stroke (CS) **(A)** and ischemic stroke (IS) **(B)**.


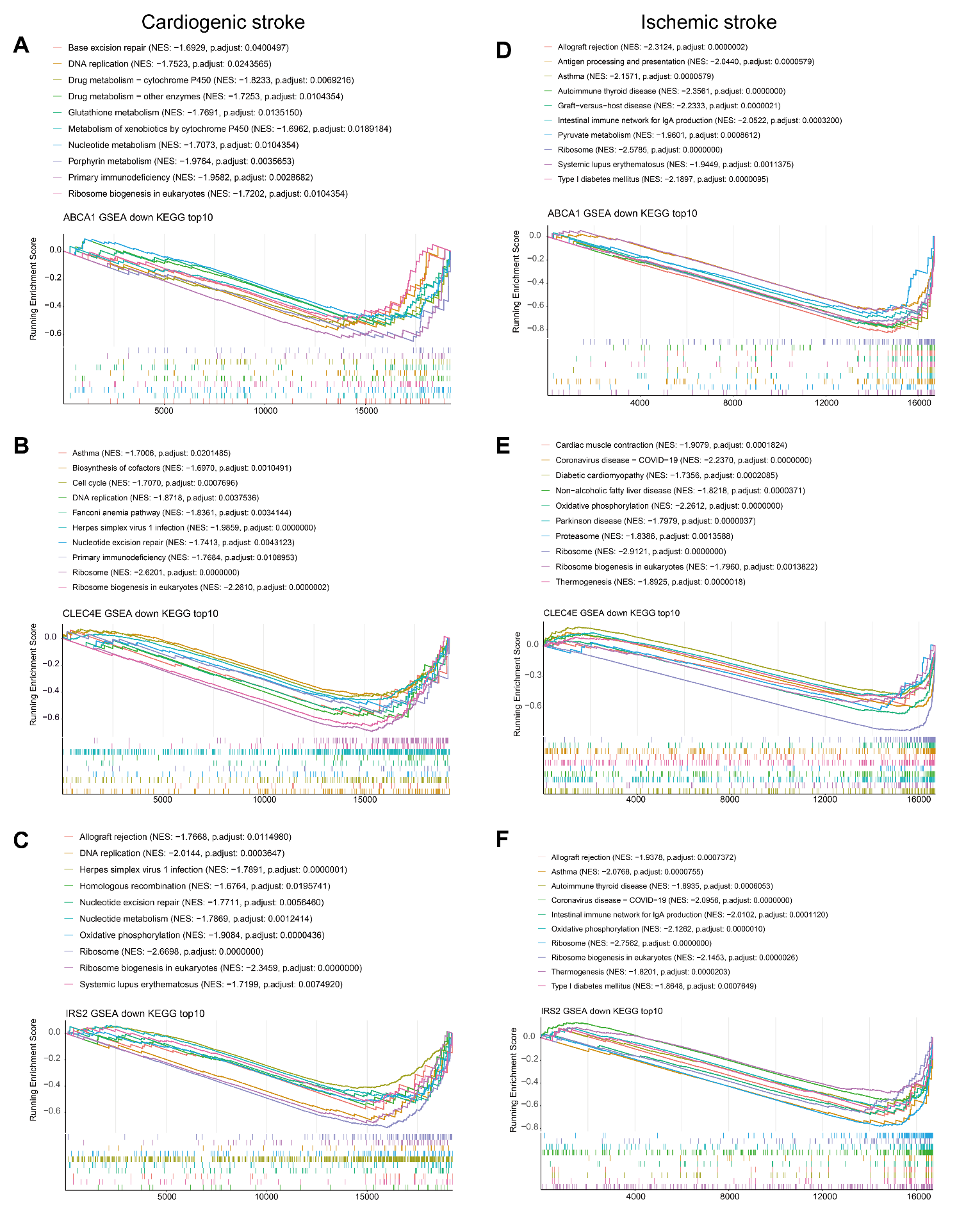


**Supplementary Figure 2. Gene set enrichment analysis.**

Top 10 inhibited KEGG pathways with ABCA1 expression in cardioembolic stroke (CS) **(A)** and ischemic stroke (IS) **(D)**; top 10 inhibited KEGG pathways with CLEC4E expression in CS **(B)** and IS **(E)**; top 10 inhibited KEGG pathways with IRS2 expression in CS **(C)** and IS **(F)**.

# Supplementary Tables

**Supplementary Table 1. Functional enrichment of the shared genes**

**Supplementary Table 2. Feature genes identified by three machine learning algorithms**

**Supplementary Table 3. Results of molecular docking**
